# *Oreocharis sihuiensis* (Gesneriaceae), a critically endangered new species from Guangdong Province, China

**DOI:** 10.3897/phytokeys.276.190710

**Published:** 2026-06-12

**Authors:** Ling-Bo Ji, Fang Wen, Xiu-Hui Jin, Yi-Da Xu, Bo-Heng Li, Bo Tang

**Affiliations:** 1 Ecological and Environmental Science and Research Institute of Zhejiang Province, Hangzhou, 310007, Zhejiang, China Guangxi Key Laboratory of Plant Conservation and Restoration Ecology in Karst Terrain, Guangxi Institute of Botany, Guangxi Zhuang Autonomous Region and Chinese Academy of Sciences Guilin China; 2 Key Laboratory of Ecological Environmental Damage Control and Value Transformation, Hangzhou, 310007, Zhejiang, China National Gesneriaceae Germplasm Resources Bank of GXIB (NGGRB), Gesneriad Committee of China Wild Plant Conservation Association (GC), Gesneriad Conservation Center of China (GCCC), Guangxi Institute of Botany, Guilin Botanical Garden, Guangxi Zhuang Autonomous Region and Chinese Academy of Sciences Guilin China; 3 Guangxi Key Laboratory of Plant Conservation and Restoration Ecology in Karst Terrain, Guangxi Institute of Botany, Guangxi Zhuang Autonomous Region and Chinese Academy of Sciences, Guilin, 541006, Guangxi, China South China Botanical Garden, Chinese Academy of Sciences Guangzhou China; 4 National Gesneriaceae Germplasm Resources Bank of GXIB (NGGRB), Gesneriad Committee of China Wild Plant Conservation Association (GC), Gesneriad Conservation Center of China (GCCC), Guangxi Institute of Botany, Guilin Botanical Garden, Guangxi Zhuang Autonomous Region and Chinese Academy of Sciences, Guilin, 541006, Guangxi, China Ecological and Environmental Science and Research Institute of Zhejiang Province Hangzhou China; 5 Key Laboratory of National Forestry and Grassland Administration on Plant Conservation and Utilization in Southern China, South China Botanical Garden, Chinese Academy of Sciences, Guangzhou, 510000, Guangdong, China Key Laboratory of Ecological Environmental Damage Control and Value Transformation Hangzhou China; 6 Young Naturalist (Hangzhou) Cultural Communication Limited Company, Hangzhou, 310012, China Young Naturalist (Hangzhou) Cultural Communication Limited Company Hangzhou China

**Keywords:** Medicinal plant, morphology, new taxon, *Oreocharis cotinifolia*, *Oreocharis dayaoshanioides*, taxonomy

## Abstract

*Oreocharis sihuiensis***sp. nov**. (Gesneriaceae) is described from Guangdong Province, China. Morphologically, the flower shape of this new species is similar to that of *O. dayaoshanioides*, but it can be distinguished by its leaf blade being adaxially densely pubescent, with hairs 0.2–0.5 mm long (*vs*. adaxially sparsely villous to villous, with hairs longer than 1 mm), lobes of the corolla upper lip subrounded and apex rounded (*vs*. broadly ovate to orbicular-ovate and apex acute), 3 staminodes (*vs*. absent or 2), disc margin cleft (*vs*. subentire), shorter filaments (ca. 6.0 mm long *vs*. 8.0–12.0 mm long), and shorter capsules (ca. 1.0 cm long *vs*. ca. 2.0 cm long). Considering its scarce number of individuals, and the presence of severe human disturbance, we preliminarily assess the new species as ‘Critically Endangered’ (CR) according to IUCN Red List Categories and Criteria.

## Introduction

The genus *Dayaoshania* W.T.Wang (Wang 1983, 1990), was established based on a distinctive species, *D. cotinifolia* W.T.Wang (1983), which was described from the Dayao Mountain, Jinxiu County, Guangxi, China. This species possesses a butterfly-shaped corolla with highly distinctive morphology. For a long time, this monotypic genus was considered a primitive lineage of Gesneriaceae due to its stamens adnate near the base of the corolla and bilocular, parallel, non-divaricate anthers (Wang 1983). However, this conclusion was not supported by systematic evidence (Möller et al. 2011). Consequently, it holds significant scientific value for investigating the phylogenetic relationships within Gesneriaceae (Wei 2010). Furthermore, *D. cotinifolia* has a narrow distribution range and few wild individuals, thus being listed as a National Key Protected Wild Plant of Category II in China (National Forestry and Grassland Administration & Ministry of Agriculture and Rural Affairs 2021).

With the continuous advancement of phylogenetic research on Gesneriaceae, the classification of Asian Gesneriaceae has undergone substantial revisions. Subsequent studies further confirmed that *Oreocharis* Benth. s.s. and a series of morphologically more or less similar genera together form a monophyletic group (Möller et al. 2011; Weber et al. 2013). Integrating morphological characteristics, the genus was expanded to include 10 genera, encompassing *Dayaoshania* as well as *Ancylostemon* W.G.Craib (1919), *Bournea* Oliv. (Hooker 1893; later restored to generic rank by Chen et al. 2020, but subsequently re-subsumed into *Oreocharis* s.l. by Kong et al. 2022), *Briggsia* W.G.Craib (1919), *Deinocheilos* W.T.Wang (1986), *Isometrum* W.G.Craib (1919), *Opithandra* B.L.Burtt (1956), *Paraisometrum* W.T.Wang (Weitzman et al. 1997), *Thamnocharis* W.T.Wang (1981), and *Tremacron* W.G.Craib (1918).

In August 2025, when we were browsing videos on the Chinese short-video app, namely ‘Douyin’ (the Chinese version of TikTok), a video about a flowering Gesneriaceae species in its native habitat attracted our attention. Morphologically, this species exhibits typical characteristics of former *Dayaoshania*, which is now merged into *Oreocharis* s.l., but its white flowers and subrounded upper corolla lobes subequal in length and width and clearly differ from those of the two known species of *Dayaoshania* type in *Oreocharis*. After contacting the videographer, we successfully collected wild individuals of this plant. Following careful dissection and comparison with two other known species of *Dayaoshania*, we concluded that it represents an undescribed new species, which is described herein.

## Materials and methods

Morphometric characters were measured on 10 mature living individuals sampled across distinct microhabitats within the only known population (including sun-exposed/shaded, and wetter/drier microsites). All measurements were taken using digital calipers, with each character measured three times per individual to minimize observation error, and mean values are reported. Voucher specimens were deposited in the Herbarium of South China Botanical Garden, Chinese Academy of Science (IBSC). Morphological terminology followed Beentje (2016).

## Taxonomic treatment

### Oreocharis sihuiensis

Taxon classificationPlantaeColeopteraCurculionidae

L.B.Ji & F.Wen sp. nov.

9D042DED-6CB8-54A1-9A66-0C03C59F22A3

urn:lsid:ipni.org:names:77381406-1

**Figure 1.**
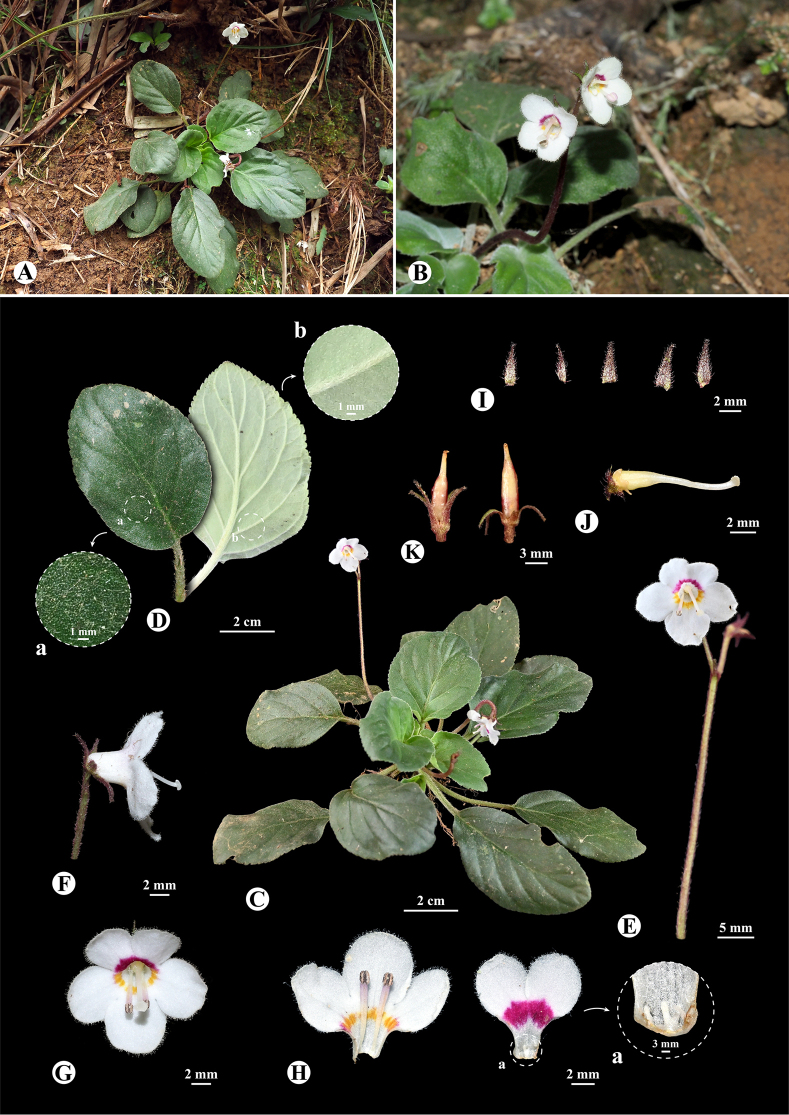
*Oreocharis sihuiensis* sp. nov. **A**. Habit; **B, C**. Flowering individuals; **D**. Leaves, adaxial (left), abaxial (right), and their partially magnified views (**a, b**. Respectively, showing indumentum); **E**. Inflorescence; **F, G**. Flowers, front and lateral view, respectively; **H**. Longitudinal section of corolla, lower lip (left), upper lip (right) and its partially magnified view (**a**. Showing indumentum and staminodes); **I**. Calyx lobes; **J**. Disc and Pistil; **K**. Capsules.

**Figure 2.**
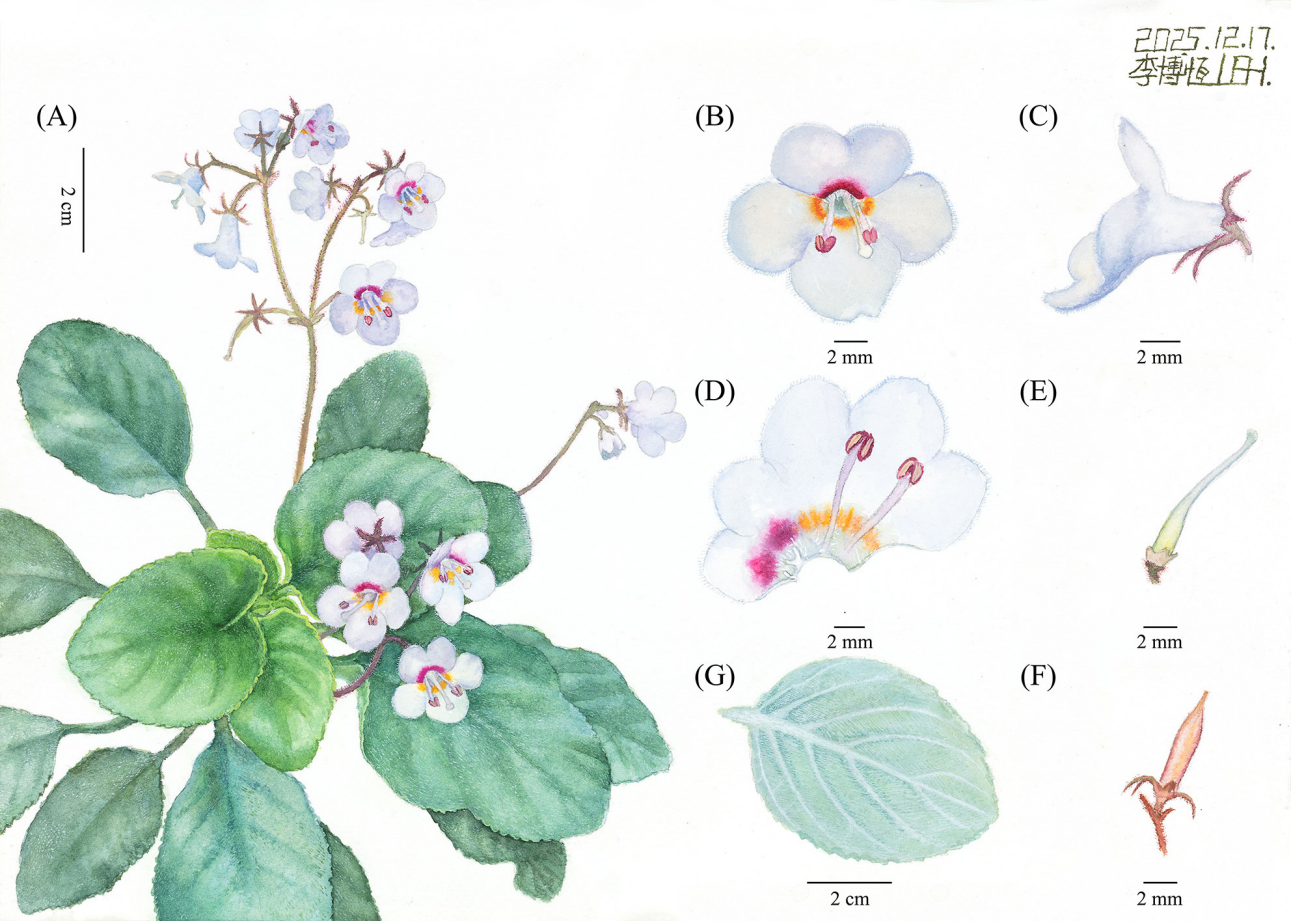
*Oreocharis sihuiensis* sp. nov. **A**. Habit; **B**. Front view of a flower; **C**. Lateral view of a flower; **D**. Longitudinal section of corolla; **E**. Disc and Pistil; **F**. Capsules; **G**. Leaf, abaxial surface.

**Figure 3.**
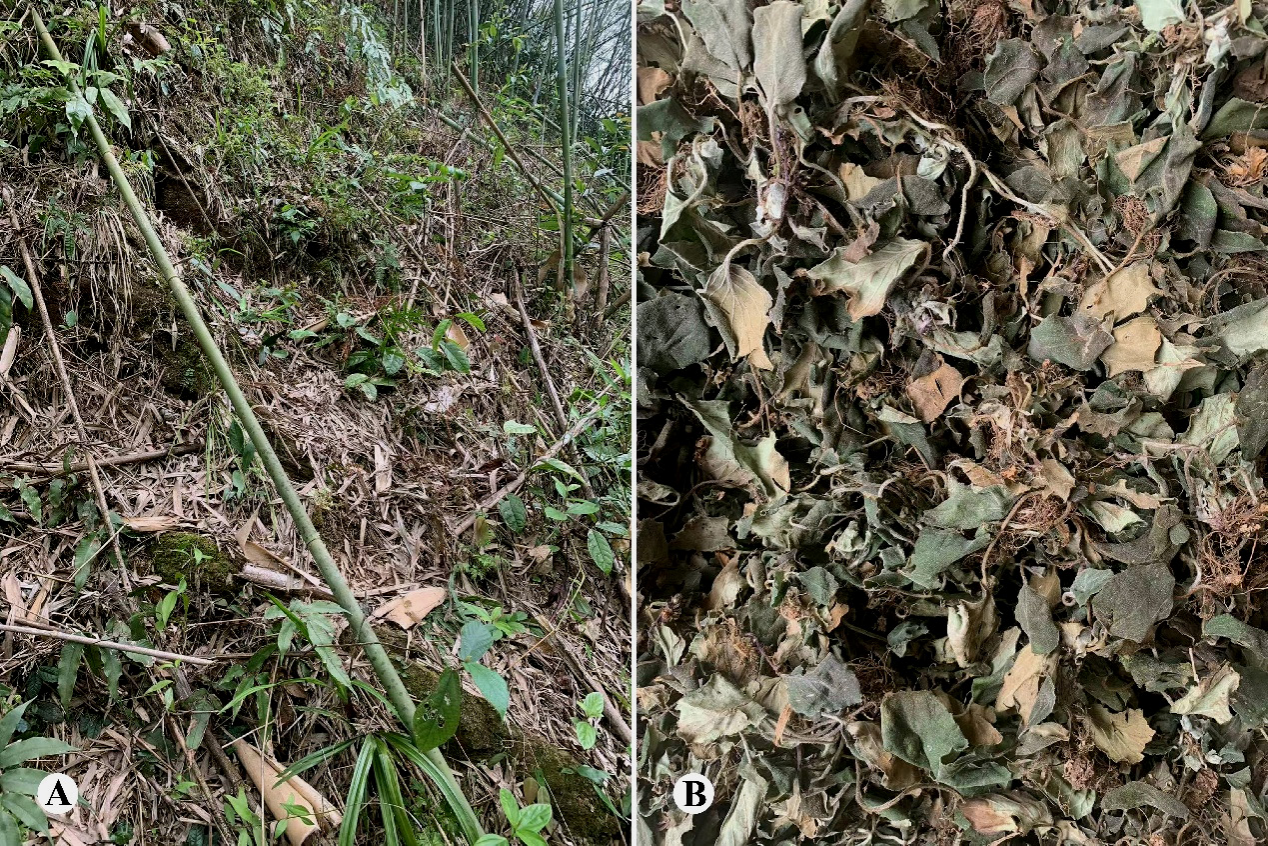
The habitat destruction and human-induced collection of *Oreocharis sihuiensis* sp. nov. **A**. Habit destruction, showing the felling of bamboo forest; **B**. Human-collected dried plants for medicinal use.

**Figure 4.**
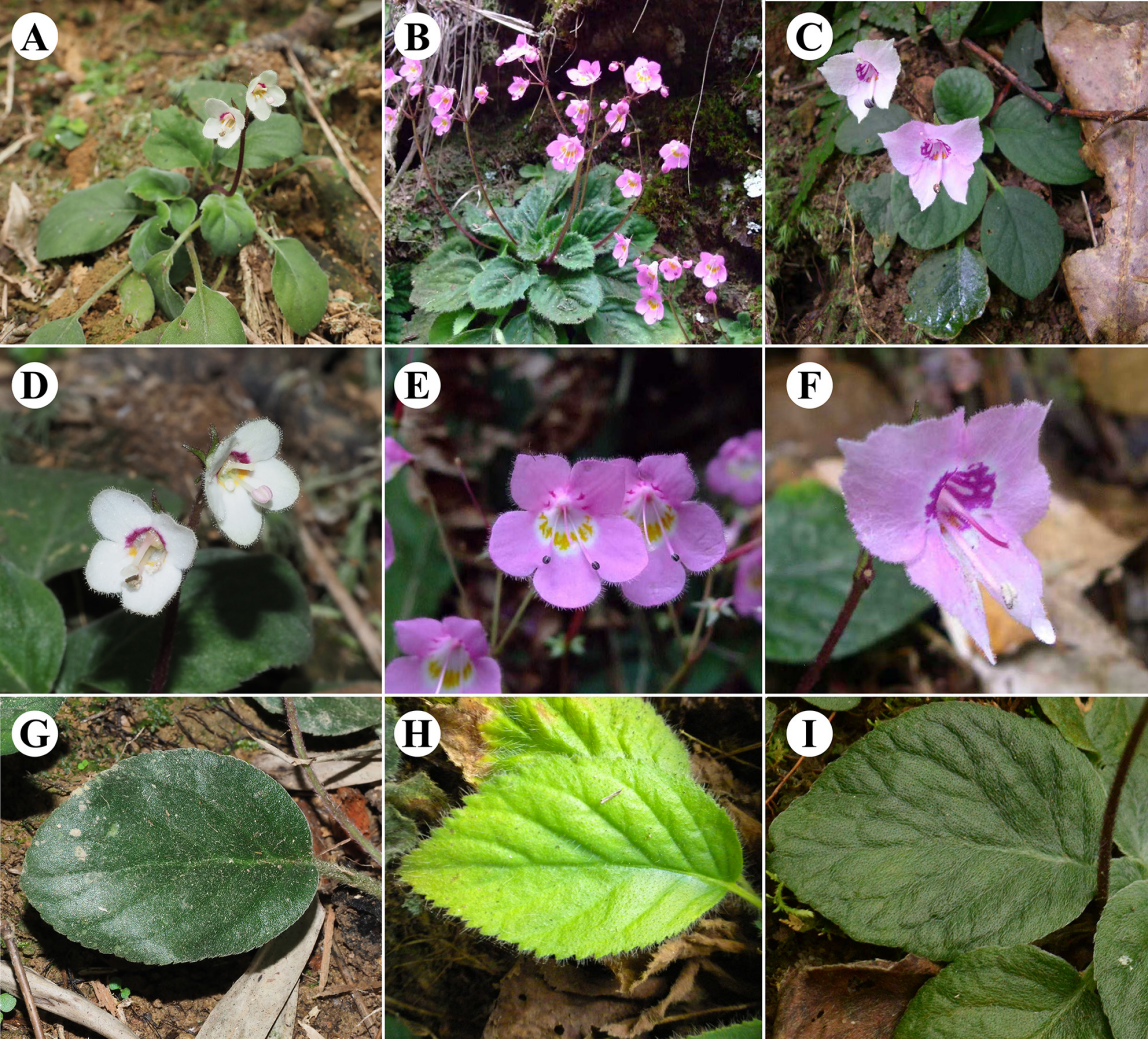
Comparison of all 3 known *Dayaoshania* type species of *Oreocharis* s.l. **A, D, G**. Flowering plants, flowers and leaves of *O. sihuiensis* sp. nov.; **B, E, H**. Flowering plants, flowers and leaves of *O. dayaoshanioides*; **C, F, I**. Flowering plants, flowers and leaves of *O. cotinifolia*.

Figs 1, 2, 3, 4A, D, G

#### Type.

China • Zhejiang Province: Yiwu City, vouchers were made from cultivated plants at Dongya Jifeng Greenhouse, 10 May 2025 (flowering), *Ling-Bo Ji, JLB2025001* (Holotype: IBK!), introduced from Guangdong Province: Sihui City, Shigou Town, 28 April 2025.

#### Diagnosis.

A species similar to *Oreocharis dayaoshanioides* Yan Liu & W.B.Xu (in Liu et al. 2012), but can be distinguished from the latter by the adaxial leaf blade being densely pubescent with hairs 0.2–0.5 mm long (*vs*. sparsely to densely villous, hairs longer than 1 mm), lobes of the corolla upper lip subrounded with a rounded apex (*vs*. broadly ovate to orbicular-ovate with an acute apex), presence of 3 staminodes (*vs*. absent or 2), disc margin cleft (*vs*. subentire), filaments shorter, less than ca. 6.0 mm long (*vs*. 8.0–12.0 mm), and capsules ca. 1.0 cm long (*vs*. ca. 2.0 cm). (Table 1, together with the type species of *Dayaoshania*, now namely *Oreocharis cotinifolia*).

**Table 1.** Morphological comparison of *Oreocharis sihuiensis* and its morphologically similar taxa.

Characteristics	*O. sihuiensis*	*O. dayaoshanioides*	*O. cotinifolia*
Margin of leaf blade	Serrulate	Serrulate	Subentire to obscurely crenulate
Indumentum of leaf blade adaxially	Pubescent with hairs 0.2–0.5 mm long	Villous with hairs longer than 1 mm	Pubescent with hairs 0.2–0.4 mm long
Inflorescences	1–10-flowered, 1–3 branched	Numerous flowered (usually more than 20-flowered), 1–4-branched	1 or 2-flowered, not branched
Lobes of corolla upper lip	Subrounded, apex rounded, margin entire	Broadly ovate to orbicular-ovate, apex acute to muticous, margin entire	Broadly ovate, apex cuspidate to acute, margin lobed or subentire
Filaments	ca. 6.0 mm long, glabrous	8.0–12.0 mm long, glabrous	7.5–14.0 mm long, pubescent
Staminodes	3	Absent or 2	Absent or 2
Disc	Ring-like, margin cleft	Ring-like, margin subentire	Stellate ring-like, margin subentire
Pistil	ca. 0.9 cm long, Glabrous	1.5–1.7 cm long, Glabrous	1.0–1.6 cm long, Pubescent
Length of capsule (cm)	ca. 1.0	ca. 2.0	ca. 2.5

#### Description.

***Perennial herbs*. *Rhizomatous stem*** short. ***Leaves*** all basal, petiolate; petiole 15–55 mm long, 2–4 mm in diameter, densely white pubescent with hairs 0.2–0.5 mm long on both sides; leaf blade chartaceous, 5.0–8.0 × 3.0–5.0 cm, ovate, oval, broadly elliptic to subrounded, base broadly cuneate to subrounded, sometimes slightly oblique, apex broadly acute to subrounded, margin serrulate, densely white pubescent on both sides, and densely white villous along veins abaxially; midrib depressed adaxially and prominent abaxially, secondary veins 4–6 pairs, conspicuous abaxially. ***Inflorescences*** cymes, 1–10-flowered, 1–3-branched; peduncle 5.0–10.0 cm long, densely purple pubescent; bracts 2, linear, ca. 6.5 × 1.3 mm, apex acute or obtuse, margin entire, more or less purple pubescent abaxially. ***Pedicels*** 0.3–1.4 cm long, densely purple pubescent. ***Calyx*** 5-lobed almost to the base, lobes narrowly triangular, 3.5–4.5 × 0.8–1.5 mm, purple pubescent abaxially. ***Corolla*** white externally, white with rose red at the throat of upper corolla tube and yellow at the throat of lower corolla tube internally, pubescent externally, pubescent at the corolla tube internally; corolla tube ca. 4.5 mm long, cross-section at the entrance of corolla throat subrounded, ca. 3.0 mm in diameter, corolla tube base ca. 2.1 mm in diameter; limb 2-lipped, upper lip ca. 5.0 × 9.0 mm, bifid to nearly base, lobes ca. 4.7 × 4.8 mm, subrounded, apex rounded, margin entire, lower lip ca. 7.5 × 13.5 mm, 3-partite to nearly base, lateral lobes ca. 4.9 × 4.9 mm, rounded to broadly ovate, apex rounded, margin entire, central lobe ca. 5.8 × 6.0 mm, subrounded, apex rounded, margin entire. ***Stamens*** 2 or occasionally 1 or 3; filaments white or purplish at apex and gradually changed to yellowish at base, ca. 6.0 mm long, adnate to ca. 0.5 mm above corolla base, glabrous; anthers dorsifixed, ellipsoid, pale gray, ca. 1.2 × 0.9 mm, glabrous. ***Staminodes*** 3, white, adnate to nearly base of corolla, lateral two ca. 7.0 mm long, central one ca. 9.0 mm long, glabrous. ***Disc*** ring-like, margin cleft, yellow, ca 0.9 mm high. ***Pistil*** ca. 9 mm long; ovary ca. 3.0 mm long, ca. 1.5 mm in diameter, yellowish, glabrous or sometimes sparsely pubescent; style ca. 6.0 mm long, yellowish, glabrous; stigma bilobed, subrounded, ca. 0.4 × 0.7 mm. ***Capsules*** ca. 1.0 cm long, glabrous.

#### Distribution and phenology.

*Oreocharis sihuiensis* grows on the moist slopes or rock walls under mixed forest of evergreen broad-leaved forest and bamboo forest, at an elevation of about 100 m. It flowers from April to May and fruits from May to July.

#### Etymology.

Specific epithet is derived from the type locality, Sihui City.

#### Vernacular name.

Sì Huì Yáo Shān Jù Tái (Chinese pronunciation); 四会瑶山苣苔 (Chinese name).

#### Proposed IUCN conservation status.

Due to excessive human exploitation, fewer than 30 individuals of this species currently survive in the wild. The population is experiencing a continuing and rapid decline, with no recruitment or juvenile individuals observed in recent field surveys, indicating an extremely negative population trend. This species is known only from this single locality, and the area of occurrence is estimated to be less than 100 m^2^. The type locality is situated near a village, characterized by frequent anthropogenic disturbance and a plant community with low species diversity. The habitat quality has been deteriorating rapidly due to ongoing human activities, with an estimated rate of habitat loss exceeding 30% over the past decade. Additionally, this species is a well-known ethnobotanical resource, widely utilized by local villagers who harvest and process the plant for medicinal tea. Furthermore, the species is known to local residents by the vernacular name ‘Shi Shang Lian’, meaning ‘Lotus Born on Rock’, which frequently leads to misidentification with congeneric species—specifically, *Oreocharis benthamii* Clarke var. benthamii or *O. benthamii* var. *reticulata* Dunn, both of which are traditionally used in Guangdong folk medicine. These folk medicinal plants are all believed to be effective in treating liver ailments, which has consequently resulted in intensive over-collection and trade, severely compromising the survival of the species. Compounding this pressure, the surrounding bamboo forest is also experiencing localized human-induced felling (Fig. 3), further reducing available habitat and exacerbating habitat fragmentation. Based on our field surveys, the type locality population is severely fragmented, with only sparse, scattered individuals remaining. The total number is around 30 individuals, indicating a population that is barely clinging to survival. Therefore, the new species is under high threat. According to Criterion D and B2 a, b (ii,iii,v) of the IUCN Red List Categories and Criteria, the new species is hereby preliminarily assessed as “Critically Endangered, CR B2 a, b (ii,iii,v); D” (IUCN 2024).

#### Distribution and habitat.

This new species is currently known only from its type locality in Shigou Town, Sihui City, Guangdong Province, China. Fewer than 30 mature individuals are known to survive. It prefers shady rock outcrops in bamboo forests.

## Discussion

The initial observation of *O. sihuiensis* was made via a ‘Douyin’ video posted by a local villager. This highlights a novel pathway for taxonomic discovery: through social media platforms, the general public may unintentionally become crucial contributors to biodiversity documentation. However, this phenomenon is a double-edged sword. While it accelerates the discovery of new species, the disclosure of precise geographical coordinates in such videos or subsequent publications may increase the risk of illegal poaching and over-collection, especially for plants with horticultural appeal or medicinal value. We strongly recommend that conservation authorities and journals establish guidelines for anonymizing or generalizing locality data of critically endangered species, while still meeting scientific standards for replicability. In the present case, although the local name “Shi Shang Lian” has led to over-harvesting for medicinal tea, we have carefully managed the timing of releasing detailed locality information after the species description to avoid exacerbating the threat.

### Key to all 3 *Dayaoshania* type species of *Oreocharis* s.l. (Fig. 4)

**Table:** 

1	Leaf blade margin subentire to obscurely crenulate; pistil and stamens pubescent; disc stellate ring-like	***O. cotinifolia***
–	Leaf blade margin serrulate; pistil and stamens glabrous; disc ring-like	**2**
2	Leaf blade pubescent adaxially, with hairs 0.2–0.5 mm long; corolla externally white; filaments ca. 6.0 mm long	***O. sihuiensis***
–	Leaf blade villous adaxially, with hairs longer than 1 mm; corolla externally pink; filaments 8.0–12.0 mm long	***O. dayaoshanioides***

## Supplementary Material

XML Treatment for Oreocharis sihuiensis

## Additional information

### Artificial Intelligence (AI) use

The authors accept full responsibility for the content of the manuscript, including the disclosure of any use of AI.

No AI tools were used in the preparation of this manuscript.

### Author ORCIDs

L.-B. Ji https://orcid.org/0009-0009-2005-021X

F. Wen https://orcid.org/0000-0002-3889-8835

X.-H. Jin https://orcid.org/0009-0004-1484-6784

Y.-D. Xu https://orcid.org/0000-0001-9812-4770

B.-H. Li https://orcid.org/0009-0005-8847-6883

B. Tang https://orcid.org/0009-0008-4918-5313

### Data availability

All of the data that support the findings of this study are available in the main text.
